# Dampness and student-reported social climate: two multilevel mediation models

**DOI:** 10.1186/s12940-021-00710-5

**Published:** 2021-03-19

**Authors:** Eerika Finell, Asko Tolvanen, Juha Pekkanen, Timo Ståhl, Pauliina Luopa

**Affiliations:** 1 School of Social Sciences, Tampere University, 33014 Tampere, Finland; 2 Methodology Centre for Human Sciences, University of Jyväskylä, PO Box 35, 40014 Jyväskylä, Finland; 3 Department of Public Health, University of Helsinki, PO Box 20, 00014 Helsinki, Finland; 4 Environmental Health Unit, Finnish Institute for Health and Welfare, PO Box 95, 70701 Kuopio, Finland; 5 Department of Welfare, Finnish Institute for Health and Welfare, Biokatu 10, 33520 Tampere, Finland; 6 Department of Welfare, Finnish Institute for Health and Welfare, PO Box 30, 00271 Helsinki, Finland

**Keywords:** Indoor air quality, Class spirit, Multilevel analysis, Teacher-student relationships, Indoor environmental problems, Mould, Dampness

## Abstract

**Background:**

Little previous research has analysed the relationship between schools’ indoor air problems and schools’ social climate. In this study, we analysed a) whether observed mould and dampness in a school building relates to students’ perceptions of school climate (i.e. teacher-student relationships and class spirit) and b) whether reported subjective indoor air quality (IAQ) at the school level mediates this relationship.

**Methods:**

The data analysed was created by merging two nationwide data sets: survey data from students, including information on subjective IAQ (*N* = 25,101 students), and data from schools, including information on mould and dampness in school buildings (*N* = 222). The data was analysed using multilevel mediational models.

**Results:**

After the background variables were adjusted, schools’ observed mould and dampness was not significantly related to neither student-perceived teacher-student relationships nor class spirit. However, our mediational models showed that there were significant indirect effects from schools’ observed mould and dampness to outcome variables via school-level subjective IAQ: a) in schools with mould and dampness, students reported significantly poorer subjective IAQ (standardised β = 0.34, *p* < 0.001) than in schools without; b) the worse the subjective IAQ at school level, the worse the student-reported teacher-student relationships (β = 0.31, *p* = 0.001) and class spirit (β = 0.25, *p* = 0.006).

**Conclusions:**

Problems in a school’s indoor environment may impair the school’s social climate to the degree that such problems decrease the school’s perceived IAQ.

## Background

In many countries, children spend a large part of their day in school buildings, where indoor climate conditions can be poor [1–3]. A cumulative body of research has pointed to the importance of optimizing these conditions because they may have adverse health effects on schoolchildren [4–6]. In addition, poor indoor climate conditions may increase absentee rates and decrease academic performance [7–10].

In addition to these harms, poor indoor climate conditions may also influence social relationships in schools. Literature from various fields supports this supposition. Slow-moving environmental disasters often erode community cohesion, leading to conflict and social alienation [11–13]. The school facility literature shows that students report social disorder and teachers are less motivated to teach when schools’ physical environments are perceived to be dilapidated or in need of repair [14, 15]. Indoor air problems in workplaces are often accompanied by social conflicts and experiences of injustice [16, 17]. However, only little is known about whether and how poor indoor climate is related to students’ perception of the *social climate* in schools, although the need to study this effects is already acknowledged in the field [18]. Social climate here refers to the interpersonal relationships and teaching practices present in a specific school [19, 20]. To the best of our knowledge, only one study published in English to date has analysed this; it showed that student-perceived teacher-student relationships were worse in schools with indoor air problems than in schools without [21].

### The present study

This paper focuses on students’ perceptions of the quality of teacher-student relationships and class spirit (i.e. the classroom’s emotional climate), which are important components of student-perceived social climate [19, 22]. These components greatly influence students’ psychosocial well-being, behaviour and academic performance [23–25].

**Fig. 1:**
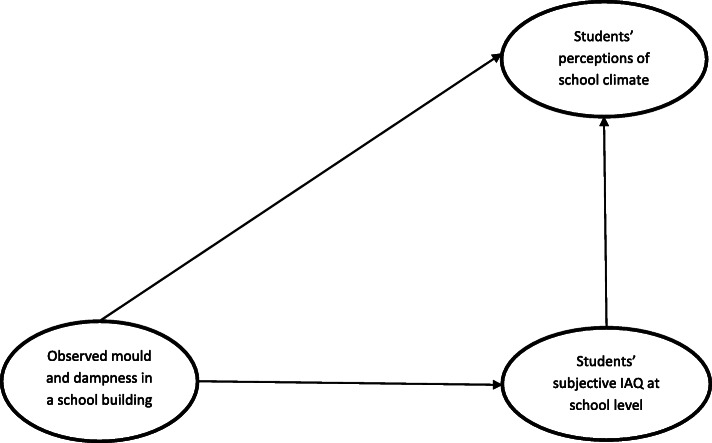
Conceptual model

Our study has two aims. First, it analyses whether a school’s observed mould and dampness is related to student-perceived teacher-student relationships and class spirit, using a large representative sample of students in Finland. Moisture damage in schools is relatively common in many countries [26], and it may have adverse health effects [5]. Second, the article analyses whether this relationship is mediated by students’ overall perception of their school’s indoor air quality (IAQ) (i.e. school-level subjective IAQ). That is, we suppose that moisture damage per se does not necessarily affect social climate. The important mediator is the effect of the school’s IAQ as perceived by its users. Previous research has shown that schools’ indoor air problems are related to subjective IAQ [27–29], and that subjective IAQ is related to student-perceived teacher-student relationships [30]. However, no previous studies have tested whether school-level subjective IAQ mediates the relationship between a school’s indoor air problems and student-perceived social climate. Our conceptual model is visualised in Fig. 1.

## Methods

### Data and participants

The *student-level* data was obtained through the School Health Promotion Study (SHP), a nationwide classroom survey. The SHP has monitored the health and well-being of Finnish adolescents since 1996, and it is conducted by the Finnish Institute for Health and Welfare (THL). Our data collection was approved by the THL’s ethical committee (THL/1704/6.02.0 1/2016).

We focused on students in the eighth and ninth grades (14–16 years old). The adolescents were informed of the aim and content of the survey, and they had the opportunity to decline to take part. Their parents and guardians were also informed. Written consent was not necessary, since the survey was conducted anonymously. The data was collected in 2017 during school lessons. In total, 84% (*N* = 730) of Finland’s lower-secondary schools participated.

The *school-level* data was obtained from the Benchmarking System of Health Promotion Capacity-Building’s (BSHPCB) data collection from comprehensive schools. This data too was collected in 2017. The BSHPCB is a nationwide benchmarking tool for local governments and schools to manage, plan and evaluate their own health promotion activities and resources in basic education. The data collection form is completed by the school’s principal together with a student welfare team. The BSHPCB is run by the THL and the data collection in basic education is done in collaboration with Finnish National Agency for Education. In total, 91% of Finland’s lower-secondary schools participated.

We included schools in our analyses using two variables from the BSHPCB. The first variable measured when the most recent inspection of the health and safety of the school environment and the well-being of the school community had been carried out. This inspection is required by Healthcare Act 1326/2010, which states that all schools in Finland should be checked every 3 years. The triennial official inspection is conducted in cooperation with the school health service, representatives of the school (e.g. the principal), representatives from the health authority, occupational healthcare, occupational health and safety, and the authorities responsible for the construction and maintenance of school buildings [31]. The inspection that focuses on building-related factors is reported in detail elsewhere [21]. For our analyses, we selected only schools where the inspection had been carried out in 2016 or 2017.

The second variable measured whether mould and dampness had been observed in the school (see section A building-related predictor). We included in our analyses the schools where a) mould and dampness had been identified during the check and the problems had not been remediated, and b) no mould and dampness had been identified during the check. We excluded from our analyses schools with fewer than 10 students (*N* = 51), students that needed special education (*N* = 89) and students who did not report their age or reported that their age was less than 14 (*N* = 340).

The final data set consisted of 25,101 students from 222 schools where both the inspection of the health and safety of the school environment and SHP were conducted.

### Measures

#### Outcome variables

*The perceived quality of teacher-student relationships* was measured by three items: ‘teachers encourage me to express my opinion in class’; ‘teachers are interested in how I am doing’; ‘teachers treat us (students) fairly’. The response scale was 1 = fully agree, 2 = agree, 3 = disagree, 4 = fully disagree. A mean rating of the items was calculated. Only if the respondent had answered all three items was the score calculated. These items have also been used in many previous studies as indicators of teacher-student relationships [30, 32]. The reliability was reasonable (Cronbach’s alpha = 0.75). The data source was the SHP.

*Class spirit* was measured by three items: ‘it’s peaceful to work in my class’; ‘the atmosphere in our class is such that I dare to express my opinion freely’; ‘the pupils in my class get along well’. The response scale was the same as above, and the mean rating was calculated similarly. These items have also been used in many previous studies as indicators of class spirit [21, 27]. The reliability was reasonable (Cronbach’s alpha = 0.68). The data source was the SHP.

#### Mediator

Our mediator was *the subjective assessment of IAQ (subjective IAQ).* It was measured by two items: ‘have any of the following things bothered you at your school during **this school year**? a) ‘Stuffy air (bad indoor air)’; b) ‘unpleasant odour’. These items were measured on a three-point scale (1 = not at all, 2 = some, 3 = a lot). A mean rating of the items was calculated. If the respondent had not answered both items, the score was not calculated. Cronbach’s alpha was 0.71. The data source was the SHP.

#### A building-related predictor

*Observed mould and dampness* was measured by one item: ‘were the following issues evaluated in the most recent inspection of the health and safety of the school environment: problems with mould and dampness?’ The response options were: 1) no data available; 2) not included in the inspection; 3) inspected, no deficiencies detected; 4) inspected, deficiencies detected but not yet corrected; 5) inspected, deficiencies detected and corrected. In this study, we included in the analyses only on the third and fourth options, and they were recoded as follows: 0 = inspected, no deficiencies detected; 1 = inspected, deficiencies detected but not yet corrected. The data source was the BSHPCB.

#### Background variables

*Gender* and *age* were used as student-level background variables only. *Fathers’ level of education* and *student-perceived teacher-student relationships* were used as both student-level and school-level background variables. Fathers’ level of education was used as an indicator of students’ socio-economic status. The response options on fathers’ education were: 1 = comprehensive school or equivalent (i.e. primary level), 2 = upper-secondary school, high school or vocational education institution (i.e. secondary level), 3 = occupational studies in addition to upper-secondary school, high school or vocational education institution (i.e. secondary level and occupational studies), 4 = university, university of applied sciences or other higher-education institution (i.e. tertiary level). All these background variables were reported by the SHP. The *school size* (i.e. number of students) reported by the BSHPCB was used as a school-level background variable only.

### Calculation

The mediation analyses were conducted by analysing two two-level linear regression path models [33]: one where the outcome measure was student-reported teacher-student relationships, and one where the outcome measure was class spirit. Multilevel analysis is required when the data is hierarchical [33]. We built the models and then analysed them using Mplus statistical software 8.0 [34]. We used full information maximum likelihood estimation (FIML) with robust standard errors (the MLR estimator in Mplus) as an estimation method. MLR is robust to moderate violations of assumptions such as non-normality [35].

We used a latent factor approach first introduced by Jöreskog [36]. In order to estimate the student-level and school-level variance in each variable in the model, their total variance was decomposed into two latent uncorrelated components by Mplus. The first latent component (i.e. student level) represented the degree students’ answers deviated from their school mean (e.g. the cluster mean of reported symptoms). The second latent component (i.e. school level) represented the degree the school mean deviated from the grand mean [34, 37].

We started by analysing the intraclass correlations (ICC) by using a null model. In a null model only the outcome variable without any predictors is inserted in the model. It is used to estimate the variance between student and school levels and the ICC [33]. The ICC reports the proportion of the variance belonging to the school level [33]. Then we analysed design effects (DEFF) of each variable. The DEFF reports how much larger a variable’s sampling variance is from the mean than would be the case if the sample had been drawn from a simple random population [38]. When a DEFF is greater than 1.1 and the researcher is interested in estimating the effects of group-level predictors, multilevel modelling is needed [39]. The DEFF can be estimated as a function of the ICC and average cluster size [38].

**Fig. 2:**
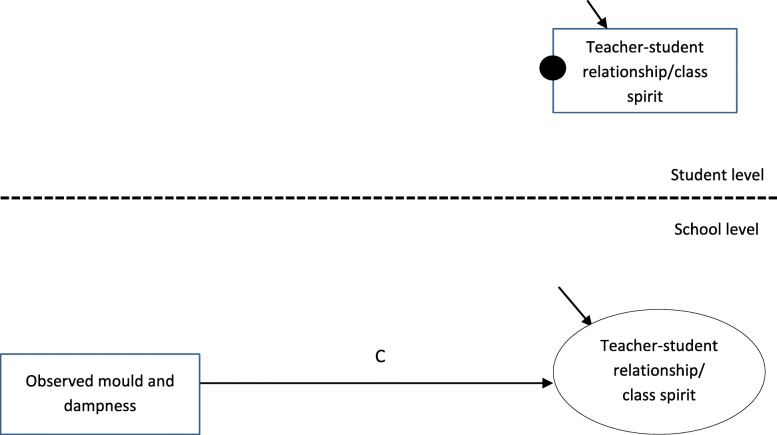
Statistical model of the total effect between observed mould and dampness and social climate outcome variables. The solid black circle corresponds to random intercept. The small arrows correspond to residual variance. Observed variables are represented by rectangular boxes and latent variables by ellipses

**Fig. 3:**
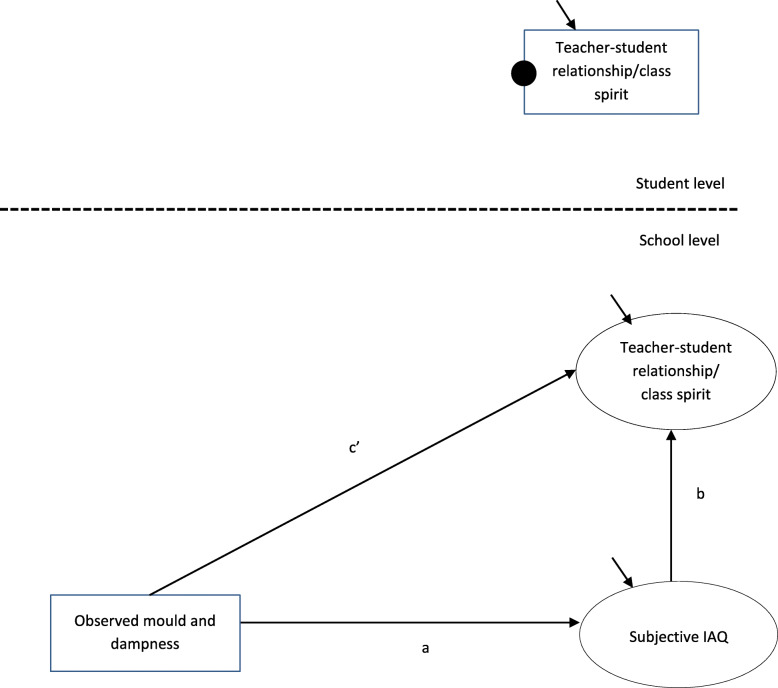
Statistical model of the direct and indirect effects between observed mould and dampness and social climate outcome variables. The solid black circle corresponds to random intercept. The small arrows correspond to residual variance. Observed variables are represented by rectangular boxes and latent variables by ellipses

Then, we estimated the *total*, *direct* and *indirect* effects of our two mediational models. The *total effect* refers to the relationship between the predictor (i.e. observed mould and dampness) and the outcome variable (i.e. teacher-student relationships or class spirit) when the mediator (i.e. subjective IAQ) is not controlled. In Fig. 2, the total effect is represented by path *C*. A significant total effect is not required when testing mediational models [40]. The *direct effect* refers to the relationship between the predictor and the outcome variables when the mediator is controlled. In Fig. 3, the direct effect is represented by path *c*’. The *indirect effect* is the product of path *a* multiplied by path *b* (see Fig. 3)*.* In our model, the independent variable and the mediator were at school level, and the outcome variable was at student level – a so-called 2–2-1 design [41]. If one variable is a school-level variable, the *indirect effect* exists at the school level [42]. The analyses were conducted according to a syntax based on articles by Preacher, Zyphur and Zhang [42] and Preacher, Zhang and Zyphur [43]. The syntax is available online at http://quantpsy.org/medn.htm.

We report both unadjusted and adjusted models. In the adjusted model, we used fathers’ education, age and gender as both student-level and school-level variables. School size and observed mould and dampness were included only at the school level. All continuous predictors and background variables were centred by their grand means.

Finally, we counted the Monte Carlo confidence intervals to assess the significance of the indirect effects. These intervals accurately reflect the asymmetric nature of the sampling distribution of an indirect effect [43]. This type of analysis has been shown to be superior to the Sobel test [44]. For helping interpretation, we present the between-level standardised coefficients of direct and total effects. To report the effect size of indirect effects, we partially standardised their regression coefficients by dividing the indirect estimates by the between-level variance of the outcome variable [45]. R^2^ was used as an indicator of explained variance. Mplus provides separate R^2^ for the student and school levels [46].

### Missing values

The number of missing values varied between the variables. Age, subjective IAQ and observed mould and dampness had the lowest percentages of missing values (0%), and socio-economic status had the highest (12%). Values were assumed to be missing at random [47]. In such cases FIML is a recommended method for handling missing data, because it uses all available data for estimation and produces unbiased parameter estimators [47].

## Results

The descriptives of all variables are reported in Table 1. About 30% of the students (*N* = 7398) studied in schools with observed mould and dampness.

**Table 1:** Descriptives of background variables, predictors and outcome variables from the raw data by indoor environment context

	Schools without dampness and mould (*N* = 162)			Schools with dampness and mould (*N* = 60)			X^2^/F-Test	*P*-value
	Mean (SD) / %	Min.–max.	N	Mean (SD) / %	Min.–max.	N		
Subjective IAQ	1.83 (0.62)	1–3	17,474	1.95 (0.62)	1–3	7312	207.02^a^	< 0.001
Teacher-student relationships	2.27 (0.61)	1–4	17,276	2.28 (0.61)	1–4	7235	0,61^a^	0,435
School spirit	2.06 (0.60)	1–4	17,360	2.05 (0.58)	1–4	7280	1,66^a^	0,198
Gender (female %)	51		8955	52		3876	6.55	0.010
Age (years)	14.86 (0.72)	14–18	17,703	14.84 (0.72)	14–18	7398	5.31^a^	0.021
Father’s education								
Primary level	9		1402	9		610	3.95	0.267
Secondary level	34		5203	32		2127		
Secondary level and additional education	22		3442	22		1445		
Tertiary level	35		5432	36		2375		
School size (students per school)	398.41 (222.15)	62–1032	162	425.37 (206.78)	28–1013	60	0.67 ^a^	0.414

^a^F-test

First, the null models were analysed [33]. The within and between variance, ICC and DEFF of outcome variables and subjective IAQ are reported in Table 2. Although the outcome variables’ variance between schools was only 3–4% of the total variance, their DEFF was strong. For example, the DEFF of 4.6 indicates that the sampling variance of the mean was almost five times larger than if the student sample had been drawn from a simple random population (see Table 2). The pairwise correlations of the main variables are reported in Table 3. Observed mould and dampness was not correlated with the outcome variables.

**Table 2:** Variance within and between schools, intraclass correlation and design effect of outcome variables and subjective IAQ (*N* = 24,511–24,786 students, 222 schools)

	σ^2^_W_	σ^2^_B_	ICC	DEFF
Student-teacher relationships	0.357	0.014	0.038	5.16
Class spirit	0.343	0.012	0.033	4.63
Subjective IAQ	0.347	0.045	0.116	13.84

**Table 3:** Correlation coefficients between main variables at student and school levels, estimated using full information maximum likelihood estimation with robust standard errors (*N* = 24,958–25,101 students, 222 schools)

	Student level		School level		
	Teacher-student relationships	School spirit	Observed mould and dampness	Teacher-student relationships	School spirit
Observed mould and dampness			1		
Teacher-student relationships	1		0.01	1	
School spirit	0.29***	1	−0.03	0.47***	1
Subjective IAQ	0.29***	0.15***	0.36***	0.27**	0.22**

*p < 0.05, **p < 0.01, ***p < 0.001

Table 4 reports the total, direct and indirect effects of the unadjusted and adjusted mediational models between *observed mould and dampness* and *teacher-student relationships*. The total and direct effects between observed mould and dampness and the outcome measure were not significant. Nevertheless, there was a significant indirect path via school-level subjective IAQ: a) in schools with observed mould and dampness, students reported significantly worse subjective IAQ than in schools without such problems (in the adjusted model, the effect size was 0.8, indicating a large effect [48]; b) the worse the subjective IAQ at the school level, the worse the student-perceived teacher-student relationships. The school-level subjective IAQ fully mediated the effect between observed mould and dampness and student-reported teacher-student relationships. The partially standardised indirect effect in the adjusted model was 0.25, which indicates a small effect [48].

**Table 4:** Unstandardised and standardised betas of the total, direct and indirect effects of the mediational models at the school level (*N* = 222)

	Unadjusted model			Adjusted model		
	Beta^a^	Stand. beta	95% CI	Beta^a^	Stand. beta	95% CI
**Teacher-student relationships**						
*Total effect*	0.003	0.011	−0.039–0.044	0.00	0.00	−0.047–0.046
*Direct effects*						
Observed mould and dampness → Poor teacher-student relationships	−0.026	−0.098	− 0.069–0.017	−0.029	− 0.108	−0.075–0.017
Observed mould and dampness → Poor subjective IAQ	0.173***	0.361	0.114–0.233	0.165***	0.343	0.096–0.234
Poor subjective IAQ → Poor teacher-student relationships	0.166**	0.297	0.070–0.262	0.175**	0.312	0.075–0.274
*Indirect effect*	0.029**	0.245^b^	0.011–0.050 ^c^	0.029**	0.245^b^	0.011–0.053 ^c^
R^2^	0.077			0.161		
**Class spirit**						
*Total effect*	−0.007	−0.028	−0.042–0.028	−0.005	−0.020	− 0.042–0.033
*Direct effects*						
Observed mould and dampness → Poor class spirit	−0.030	−0.126	− 0.069–0.008	−0.026	− 0.105	−0.066–0.014
Observed mould and dampness → Poor subjective IAQ	0.172***	0.359	0.113–0.232	0.165***	0.344	0.097–0.234
Poor subjective IAQ → Poor class spirit	0.136**	0.268	0.045–0.226	0.128**	0.251	0.037–0.219
*Indirect effect*	0.023**	0.210^b^	0.008–0.041^c^	0.021**	0.192^b^	0.006–0.038 ^c^
R^2^	0.064			0.159		

^a^Unstandardised beta. ^b^Partially standardised beta. ^c^Monte Carlo confidence intervals. **p < 0.01, ***p < 0.001

Table 4 also reports the total, direct and indirect effects of the unadjusted and adjusted mediational models between *observed mould and dampness* and *class spirit*. The total and direct effects between observed mould and dampness and the outcome measure were not significant. Nevertheless, there was a significant indirect path via school-level subjective IAQ: a) in schools with observed mould and dampness, students reported significantly worse subjective IAQ than in schools without such problems; b) the worse the subjective IAQ at the school level, the worse the class spirit. The school-level subjective IAQ fully mediated the effect between observed mould and dampness and class spirit. The partially standardised indirect effect in the adjusted model was 0.19, which indicates a small effect [48].

## Discussion

We found that observed mould and dampness was not directly related to student-perceived teacher-student relationships or class spirit at the school level. Instead, the indirect effects via subjective IAQ were significant: a) observed mould and dampness in school buildings was related to students’ overall evaluation of their school’s IAQ so that in schools with mould and dampness they reported worse IAQ; b) school-level subjective IAQ was related to social climate variables so that the worse the school-level subjective IAQ, the worse the student-reported teacher-student relationships and class spirit. Although these indirect effects were small, our findings are important: a school’s social climate is essential to students’ academic performance and psychosocial well-being [19, 22, 49]. Therefore, there is an urgent need to learn how schools’ indoor air problems also affect factors other than health and academic performance, which have mostly been studied in earlier studies.

To the best of our knowledge, only one previous study has analysed how schools’ indoor air problems affect their social climate [21]. This earlier study analysed a large body of school and student data from Finland that had been collected 4 years earlier than the data used in the present study. It showed a direct relationship between schools’ indoor air problems and student-perceived teacher-student relationships, whereas present results show a more complex relationship. In addition, only students with high average grades reported worse class spirit in schools with indoor air problems in the previous study. Although the outcome variables were the same in these two studies, building-related information was measured differently. In the former study it was measured by a more general item (i.e. biological agents – indoor air, mould etc.), whereas in the present study we used a more specific item focusing only on mould and dampness.

Our study suggests that building-related problems affect perceived social climate only if they decrease the subjective IAQ of the people that spend time in the building. The important task for future research is to better understand why poor subjective IAQ has this role. There are potentially multiple reasons. Poor subjective IAQ may act as a marker of severity of moisture and mould problems to building users. Previous school facility research has shown that when there are perceived problems in a school building, teachers feel less motivated to teach [14] and principals report that their ability to deliver instruction is disturbed [50]. These factors may relate to student-perceived school climate. Poor subjective IAQ may also reflect building-related problems [51] that may affect the health and sick absentee rates of both students and teachers [5, 6] and these factors may influence their social interaction too. Finally, poor subjective IAQ is often accompanied by evaluation and - if building-related problems are found - remediation processes that create noise and may require people to move to one or more temporary buildings. All these unusual conditions can produce stress for both teachers and students.

From the practical perspective, our findings point to the importance of preventing the potential social consequences of indoor air problems in schools. In addition to investigating and properly remediating school buildings, the authorities should pay attention to the psychosocial well-being of the organisation and map the need for social support. For trust-building, open, regular and factual information-sharing is essential [52], as well as developing clear and fair management and decision-making procedures.

Our study has both strengths and limitations. The strength of our study is the large and representative sample. Data sets that include building-level and student-level information from more than 200 schools are rare. This has allowed us to test multilevel mediational models, which have often been neglected because of small school-level samples. The fact that both our mediator and our outcome variables were derived from the same cross-sectional student data set is a limitation of our study. However, no alternative mediational models could be built, because there was no direct relation between observed mould and dampness and social climate variables. Of course, longitudinal data is needed to confirm these findings. Another limitation is that we did not have direct physical measurements, and thus we had to rely on principals’ reports of inspection results.

## Conclusion

Observed mould and dampness is indirectly related to student-perceived teacher-student relationships and class spirit via school-level subjective IAQ. These findings suggest that building-related problems worsen perceived social climate only if they decrease the subjective IAQ of the people that spend time in the building. This means that complaints of poor IAQ need to be taken seriously. In addition, the psychosocial consequences of delayed remediation processes should be taken into account when their urgency is being evaluated.

## Data Availability

The data that support the findings of this study are available from the Finnish Institute for Health and Welfare with its permission. Restrictions apply to the availability of these data.

## Abbreviations

BSHPCB: Benchmarking System of Health Promotion Capacity-Building
DEFF: Design effect
THL: Finnish Institute for Health and Welfare
FIML: Full information maximum likelihood estimation
IAQ: Indoor air quality
ICC: Intraclass correlation
SHP: School Health Promotion Study
